# Optimal chest compression position for cardiopulmonary resuscitation determined by computed tomography image: retrospective cross-sectional analysis

**DOI:** 10.1038/s41598-023-49486-3

**Published:** 2023-12-20

**Authors:** Nitima Saksobhavivat, Phatthranit Phattharapornjaroen, Patsorn Suksukon, Pongsakorn Atiksawedparit, Phanorn Chalermdamrichai, Ratchanee Saelee, Pitsucha Sanguanwit

**Affiliations:** 1 Department of Diagnostic and Therapeutic Radiology, Faculty of Medicine, Ramathibodi Hospital, Mahidol University, 270 Rama VI Rd., Thung Phaya Thai, Ratchatewi, Bangkok, 10400 Thailand; 2 Department of Emergency Medicine, Faculty of Medicine, Ramathibodi Hospital, Mahidol University, 270 Rama VI Rd., Thung Phaya Thai, Ratchatewi, Bangkok, 10400 Thailand; 3 Chakri Naruebodindra Medical Institute, Faculty of Medicine, Ramathibodi Hospital, Mahidol University, 111 Moo 14, Bang Pla, Bang Phli, Samut Prakan 10540 Thailand; 4 Department of Internal Medicine, Faculty of Medicine, Ramathibodi Hospital, Mahidol University, 270 Rama VI Rd., Thung Phaya Thai, Ratchatewi, Bangkok, 10400 Thailand

**Keywords:** Cardiology, Circulation

## Abstract

The objective of this study was to determine the height of optimal hand position for chest compression during adult cardiopulmonary resuscitation (CPR) from the tip of the sternal xiphoid process (TOX) along with the relative heights of the left ventricular outflow tract (LVOT) and abdominal organs among the Thai population. The retrospective cross-sectional study was conducted through a review of medical records and contrast-enhanced chest computed tomography. The total of 204 Thai patients without obvious chest deformity at Ramathibodi Hospital from January to June 2018 was included as part of a multi-regional study. The heights of the level of maximal LV width (LV_max_), LOVT, top of liver and stomach with respect to TOX were measured on midline sagittal image. Mean age and body mass index (BMI) were 59.5 years and 23.9 kg/m^2^, respectively. One hundred and one subjects (49.5%) had pulmonary diseases. Mean height of the LV_max_ from TOX was 37.7 mm, corresponding to 20% of the sternal length (SL) in the inspiration arm raised position (IAR). The adjusted height of LV_max_ from TOX in the expiration arm-down position (EAD) was 89.7 mm (48% of SL). The inter-nipple line was at 84.5 mm (45.1% of SL) from TOX on IAR. Among 178 and 109 subjects whose uppermost part of the liver and stomach were above TOX, 80.4% and 94.5% were located within the lower half of the sternum, respectively. The adjusted optimal hand position for chest compression during CPR was at approximately 89.7 mm from TOX in EAD (48% of SL). The hand position at the upper part of the lower half of the sternum is closest to the adjusted LV_max_ and has a better chance to avoid compression of intraabdominal organs.

*Trial registration* This trial was retrospectively registered on 2 February 2023 in the Thai Clinical Trial Registry, identification number TCTR 20230202006.

## Introduction

Cardiopulmonary resuscitation (CPR) guidelines have been periodically updated to improve CPR quality and clinical outcomes of patients with sudden cardiac arrest. Adequate chest compression has long been recognized as an essential factor for high-quality CPR. The mechanism of blood flow during CPR was proposed in two theories, the "cardiac pump" and “thoracic pump” theories. According to the cardiac pump theory, chest compressions serve to directly pump blood from heart into circulation, occurring concurrently with the opening of aortic and pulmonary valves and the closure of mitral and tricuspid valves. In contrast, the thoracic pump theory presumes that chest compression lead to an increase in intrathoracic pressure, thereby generating forward blood flow within intrathoracic vessels^1,2^. The primary objective of such compressions is to maintain blood flow and optimize oxygen delivery ensuring that vital organs, especially the heart and brain, are furnished with a requisite and uninterrupted supply of oxygenated blood^3–5^. In Thailand, the American Heart Association (AHA) guideline for CPR is widely adopted. According to the latest edition published in 2020, key factors include adequate rate and depth of chest compression which allows complete chest recoil between compressions, minimizing interruptions in compressions, and avoiding excessive ventilation^3^.

The 2020 edition along with the preceding 2010 edition of the AHA guideline for CPR recommended that the rescuer should compress at the lower half of the sternum^3,4^. This has been revised from the prior recommendation in the 2005 guideline which suggested that the rescuer should compress at the point where the sternum and inter-nipple line (INL) meet^6^. Theoretically, the more direct chest compression to the left ventricle (LV) should produce the more stroke volume of the heart^7^. However, there is insufficient evidence in the current literature regarding the proper hand position for chest compression and clinical outcomes, particularly in the Asian population.

The aforementioned researches reflect the inconclusive findings of the proper locations of the hand position during CPR. In addition, the differences between ethnicity and body configuration could possibly affect the accuracy of the hand position. Therefore, this study aims to determine the optimal anatomical position that can maximize the compression of the left ventricle while avoiding damage to abdominal organs through CT image analysis.

## Materials and methods

### Study design and setting

This retrospective cross-sectional study was conducted after the approval of The Committee on Human Rights Related to Research, Faculty of Medicine, Ramathibodi Hospital, Mahidol University review board using the data at Ramathibodi Hospital, Mahidol University, Bangkok, Thailand from January 2018 to June 2018. Ramathibodi Hospital is a medical school with 1,300 beds, 5,000 OPD visits per day, and approximately 60,000 emergency room visits per year.

### Study participants

Consecutive participants who underwent contrast-enhanced chest CT scans between January and June 2018 were reviewed. The inclusion criteria were fifteen years old or older Thai citizen (by ID card) on the day of CT scanning. The patients who had at least one of chest wall anomalies (pectus excavatum, pectus carinatum, severe kyphosis and scoliosis), obvious mediastinal shift, obvious cardiac abnormality, dextrocardia and situs inversus, and those whose bilateral nipples or normal position of abdominal organs were not demonstrable were excluded. Informed consent was waived by The Committee on Human Rights Related to Research, Faculty of Medicine, Ramathibodi Hospital, Mahidol University as the data were retrospectively collected and were anonymous.

### Variables

The variables under investigation were anatomical landmarks. These landmarks included the skin landmark position denoting the maximal width of the left ventricular base (referred to as LV_max_), the skin landmark position of the left ventricular outflow tract (LVOT), and the skin landmark positions corresponding to the uppermost regions of the liver and stomach, as discerned from a chest CT.

### Data collection

The electronic medical records and radiology registration were reviewed. Demographic data, i.e., age, gender, weight, height, body mass index (BMI), and history of underlying diseases were extracted from medical records. Patsorn S, a 3^rd^ year diagnostic radiologist in training, was responsible for extracting and entering the data. NS (an emergency radiologist with more than 8 years experiences in emergency imaging) and PA (an emergency physician with more than 5 years experiences in emergency care and resuscitation) were responsible for confirmation of the data input results.

### CT acquisition and measurement

The CT scans were performed using a 64-detector row spectral CT scanner (IQon Spectral CT, Philips Healthcare, Best, The Netherlands), with standard institutional scanning parameters as follow: 120 kVp, 50–100 mAs, auto mA, 0.4–0.5 s rotation time, pitch 1.203–1.234, 2-mm slice thickness, and 2-mm intervals.

All CT images were reviewed on the Picture Archiving and Communications System (PACS), using a DICOM Conformance (Synapse version 3.2.0, FUJIFILM Medical Systems USA’s Synapse® PACS System, USA). All measurements and data collection were performed by one of the co-authors (Patsorn S). Before actual data collection, 35 pilot cases were used for estimating intraclass correlation coefficient (ICC) between the radiology trainee (Patsorn S) and a certified radiologist (NS). ICC and their 95% confidence interval (CI) of measuring sternal length, the height from TOX to INL, LV_max_, LVOT, and liver were 0.89 (0.96, 0.99), 0.98 (0.96, 0.99), 0.96 (0.93, 0.98), 0.92 (0.84, 0.96) and 0.98 (0.96, 0.99), respectively. These indicated excellent reliability between 2 measurers.

**Figure 1:**
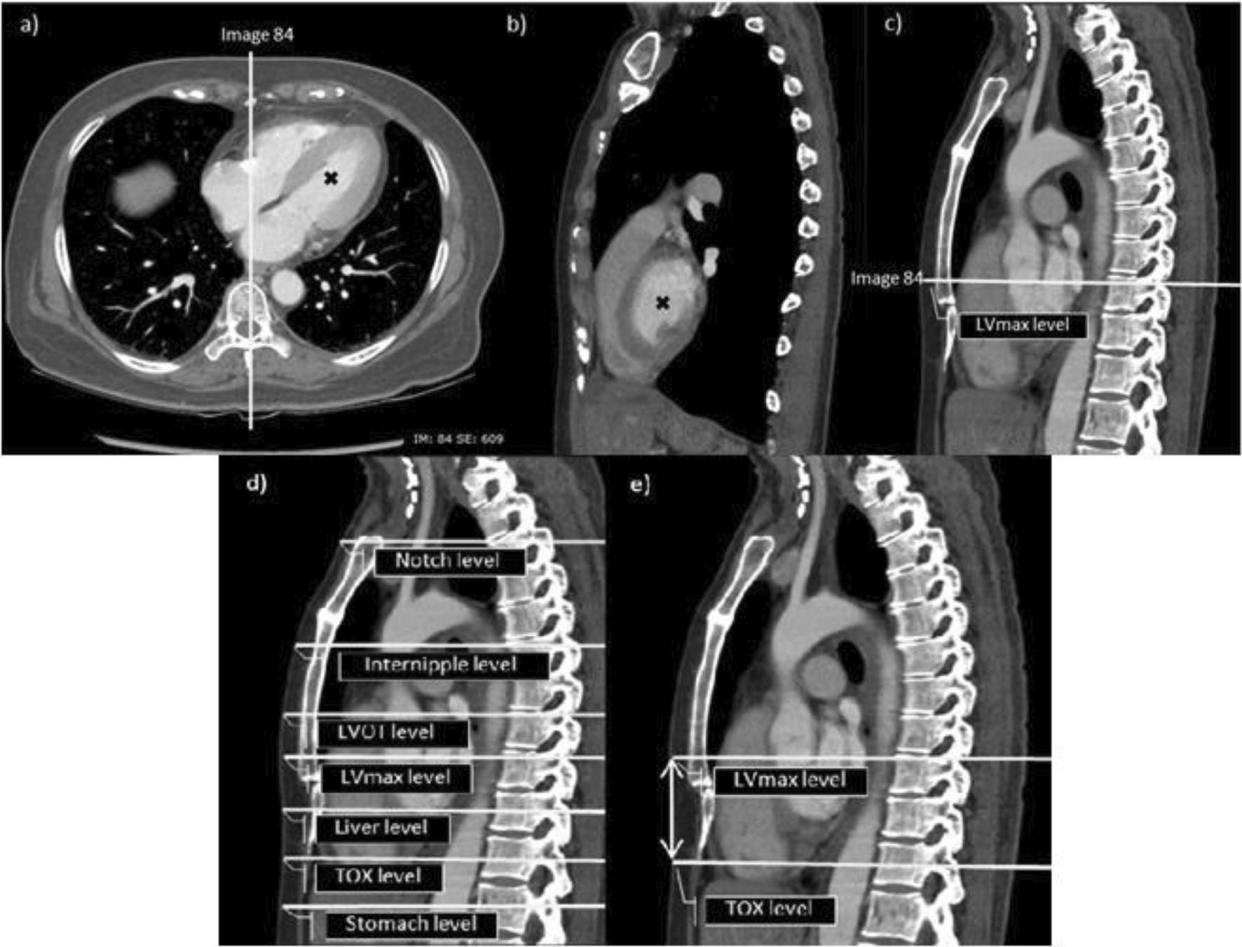
Computed tomography scan demonstrating axial (**a**), sagittal image at the left ventricular base (**b**, **c**) and midline sagittal image (**d**, **e**); (**a**) and (**b**): The level of maximal width of the left ventricular base (LV_max_) was selected using axial and sagittal images. (**a**) and (**c**): The LV_max_ level is located in midline sagittal plane using Interlink function. (**d**) The level of each reference body landmarks was identified on midline sagittal image and reference lines were drawn. (**e**) Measurements of the height from tip of xiphoid process to LV_max_ level were done at the points of overlying skin. LV_max_: The level of maximal width of the left ventricular base, LVOT: left ventricular outflow tract, TOX: Tip of xiphoid (Picture by NS. and Patsorn S).

Image analysis were performed using the methods as follow. First, the level of maximal width of the left ventricular base (LV_max_) is selected using axial and sagittal CT images. Then, the level of LV_max_ was located on midline sagittal plane using interlink function on PACS. A horizontal line corresponding to the axial level of LV_max_ is drawn on midline sagittal image and labeled as “LV_max_ level” (Fig. 1). Second, the same method is used to locate the INL, LVOT, top of the liver and stomach in the axial planes and the corresponding horizontal lines were drawn in midline sagittal image and labeled (Fig. 1). Third, we measured the distance between the skin overlying the tip of xiphoid process (TOX) level to the point of the skin which overlies each of the previously labeled levels. Fourth, we calculated the proportions of the heights from TOX to the INL, LV_max_, LVOT, adjusted LV_max_, and adjusted LVOT with respect to the sternal length (SL). Proportion of the parameters of interest compared to the sternal length are described in Supplement 1.

The heights of the LV_max_ and LVOT from TOX were measured on the CT scans which were performed during inspiration with arm raised position. These measured values were adjusted for expiration with arm down position (EAD) as found in patients with cardiac arrest by adding 52 mm to each based on the prior study by Kwon et al.^8^.

Based on Lee et al. ’s study in Korea^9^ the mean distance from TOX to LV_max_ was 27.7 mm (SD 13.3). Our sample size was determined by an infinite population mean Eq.^10^ $z^{2}_{{1 - \frac{\alpha }{2}}} \left( {\frac{{\sigma^{2} }}{{d^{2} }}} \right)$, where n is sample size, *σ* is standard deviation of mean distance from TOX to LV_max_, d is error, and α error is 0.05. Therefore, this study required at least 170 subjects with a d of 2 and α-error of 0.05. Finally, the target sample size was increased by 20% (204 subjects) for considering missing rate.

### Statistical analysis

Categorical data were described as number and percentage, whereas, continuous data were described as mean (SD) or median and interquartile range (IQR), based on their distribution. Comparison of categorical data were performed by Chi^2^ or Fisher exact tests. Student t test and Wilcoxon ranked sum test were used for comparison of continuous data with normal and non-normal distribution, respectively. All statistical analysis was performed using STATA version 15 software (Stata Corp 2017. Stata Statistical Software: Release 15. College Station, TX: Stata Corp LLC). *P*-values of less than 0.05 was considered statistically significant.

### Ethics approval and consent to participate

This study was approved by The Committee on Human Rights Related to Research, Faculty of Medicine, Ramathibodi Hospital, Mahidol University, Bangkok, Thailand (COA. MURA2019/574 Date July 07, 2019). The study was performed in accordance with the ethical standards as laid down in the 1964 Declaration of Helsinki and its later amendments or comparable ethical standards. Informed consent was waived as the data were retrospectively collected and were anonymous.

## Results

**Figure 2:**
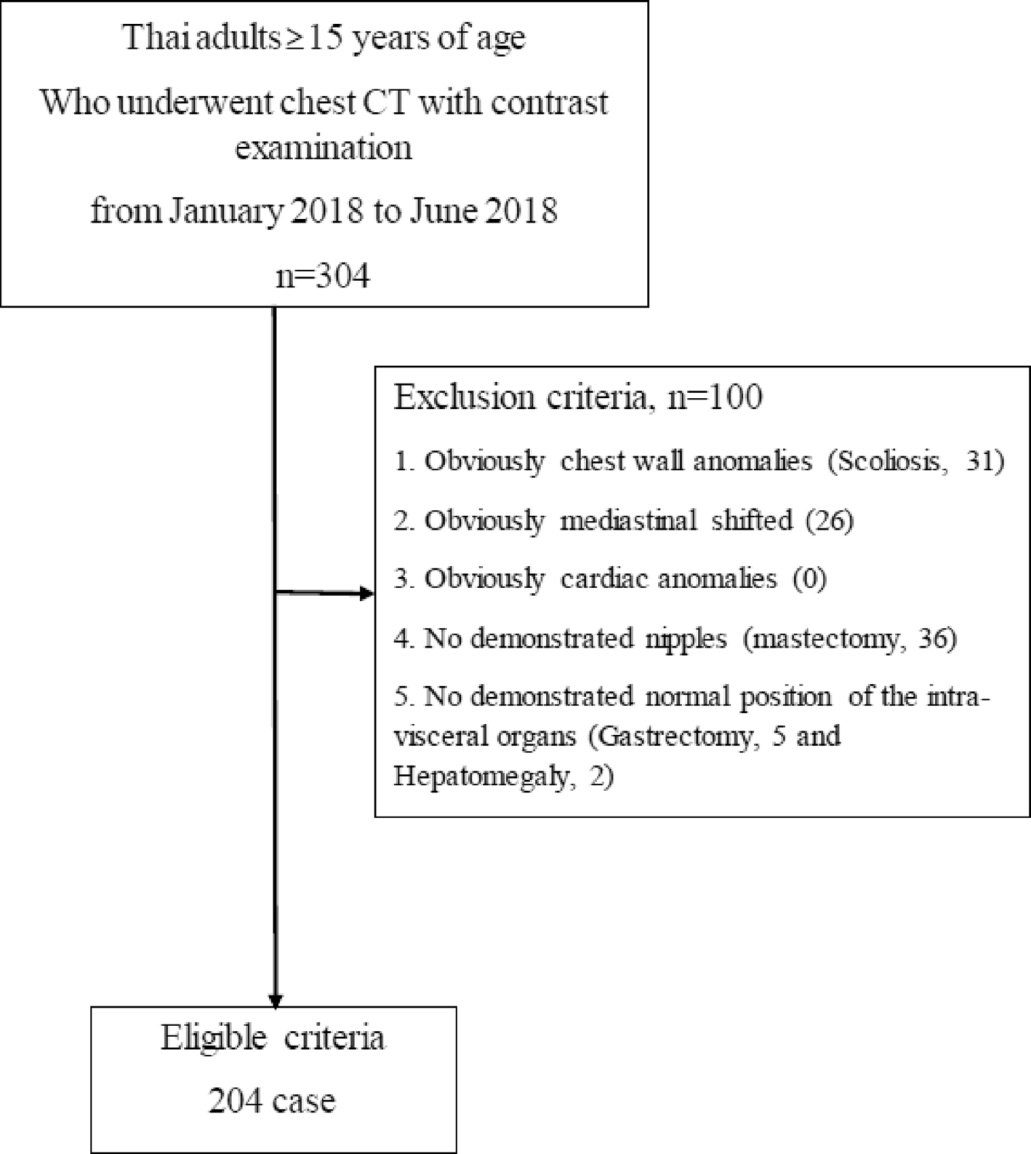
Flow chart of study population.

Three hundred and four consecutive patients who underwent contrast-enhanced chest CT at Ramathibodi Hospital were enrolled starting in January 2018. One hundred patients were excluded by the described exclusion criteria (Fig. 2). Of these, 31 patients had chest wall anomalies (scoliosis). Twenty-six patients had markedly shifted cardiac position from the midline. Thirty-six patients had undergone mastectomy. Seven patients had unclear locations of the abdominal organs; five of whom underwent partial gastrectomy and two underwent partial hepatectomy. Therefore, a total of 204 patients were included in the final analysis.

The characteristics of the patients are summarized in Table 1. Slightly more than half of the subjects were female 52% (106/204) with a mean age of 59.5 years ± 13.0 years. Mean BMI was 23.9 kg/m^2^ ± 4.2 kg/ m^2^. Of 101 patients with lung diseases; 75 patients had lung cancer or lung metastasis, 14 patients had COPD or asthma, and 12 had tuberculosis. There were 5 patients with a history of heart disease.

**Table 1:** Demographic characteristics of the study group.

Characteristics	Overall (*n* = 204)	Gender		*P* value
		Male (*n* = 98)	Female (*n* = 106)	
Age (years), mean (SD)	59.5 (13.0)	62.1 (11.2)	57.2 (13.9)	< 0.01
Weight (kilograms), mean (SD)	62.4 (12.9)	67.3 (13.2)	57.9 (10.7)	< 0.01
Height (centimeters), mean (SD)	161.3 (7.9)	166.8 (6.5)	156.1 (5.3)	< 0.01
BMI, mean (SD)	23.9 (4.2)	24.1 (4.1)	23.8 (4.3)	0.60
Heart disease, n (%)	5 (2.5)	2 (2.04)	3 (2.83)	0.72
Lung diseases, n (%)	101 (49.51)			0.79
COPD/Asthma	14 (6.9)	8 (8.16)	6 (5.66)	
Lung cancer/metastasis	75 (36.8)	33 (33.67)	42 (39.62)	
Tuberculosis	12 (5.9)	6 (6.12)	6 (5.66)	

SD: standard deviation, BMI: body mass index, COPD: chronic obstructive pulmonary disease.

**Figure 3:**
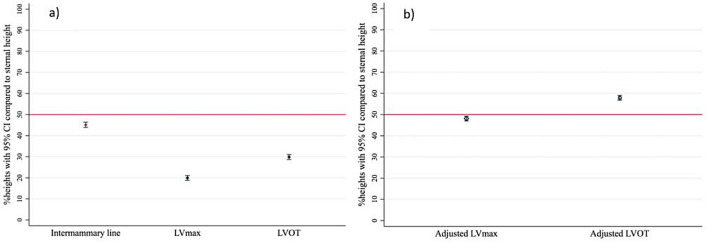
(**a**) Mean actual heights of inter-nipple line, LV_max_ and LOVT with 95% CI compared to sternal length with. (**b**) Mean adjusted LV_max_ and adjusted LOVT with 95% CI compared to sternal length. LV_max_: The level of maximal width of the left ventricular base, LVOT: left ventricular outflow tract, CI: confidence interval.

The heights from the tip of the xiphoid process to each body landmark of interest during inspiration with arm raised position (IAR) on contrast-enhanced chest CT study are shown in Table 2. Mean sternal length was 186.7 mm ± 19.6 mm. The heights of the LV_max_ and LVOT were at 37.7 mm ± 17 mm and 56.1 mm ± 17.9 mm, respectively. The proportion of these heights were 19.9% and 29.9% of the sternal length, respectively (Table 2 and Fig. 3). The height from TOX to inter-nipple line was 84.5 mm ± 19.6 mm. This was approximately 45% of sternal length. All of the direct measurements were significantly higher in male subjects. However, their proportions compared to the sternal length as shown in percentage were not significantly different among genders.

**Table 2:** The heights from tip of the xiphoid process to the reference body landmarks on CT scans during inspiration with arm raised position (IAR).

Height from TOX (mm), mean (SD)	Overall (*n* = 204)	Gender		*P* value
		Female (*n* = 106)	Male (*n* = 98)	
Sternal length (SL)	186.7 (19.6)	174.2 (13.2)	200.1 (16.2)	< 0.01
Mid sternal length	93.3 (9.7)	87.1 (6.5)	100.1 (8.1)	< 0.01
One fourth of sternal length	46.7 (4.9)	43.6 (3.3)	50.0 (4.1)	< 0.01
Inter-nipple line	84.5 (19.6)	76.6 (16.7)	92.7 (18.2)	< 0.01
% inter-nipple line to sternal length	45.1 (8.7)	43.9 (8.4)	46.5 (8.9)	0.03
LV_max_	37.7 (17.0)	34.2 (15.9)	41.5 (17.3)	< 0.01
% LV_max_ to sternal length	19.9 (8.4)	19.3 (8.4)	20.7 (8.3)	0.28
LVOT	56.1 (17.9)	52.7 (17.6)	59.7 (17.5)	< 0.01
% LVOT to sternal length	29.9 (8.5)	29.9 (8.8)	29.7 (8.2)	0.83
Uppermost liver (*n* = 178), median (IQR)	23.6 (11.9, 44.4)	22.5 (10.4, 35.6)	30.5 (13.4, 48.3)	0.20
Uppermost stomach (*n* = 109), median (IQR)	15.9 (6.9, 26.9)	15.9 (6.5, 28.2)	17.6 (7.1, 25.4)	0.74
*Adjusted LV_max_	89.7 (17.0)	86.2 (15.9)	93.5 (17.3)	< 0.01
% LV_max_ to sternal length	48.1 (8.0)	49.4 (7.6)	46.8 (8.2)	0.02
*Adjusted LVOT	107.9 (17.6)	104.7 (17.6)	111.7 (17.5)	< 0.01
% LVOT to sternal length	58.1 (8.4)	59.9 (8.1)	55.9 (8.2)	< 0.01

IAR: inspiration with arm raised position, TOX: Tip of xiphoid, SL: sternal length, SD: standard deviation, LV_max_: The level of maximal width of the left ventricular base, LVOT: left ventricular outflow tract, IQR: inter quartile range.

*Adjusted heights from tip of xiphoid process to reference body landmarks for expiration with arm down position (EAD)^8^.

There were 178 and 109 subjects whose uppermost part of the liver and stomach were above the tip of the xiphoid process. The median (IQR) height from xiphoid process to uppermost part of liver and stomach were 23.6 mm (11.9, 44.4) and 15.9 mm (6.9, 26.9), respectively. There was no significant difference among genders.

**Figure 4:**
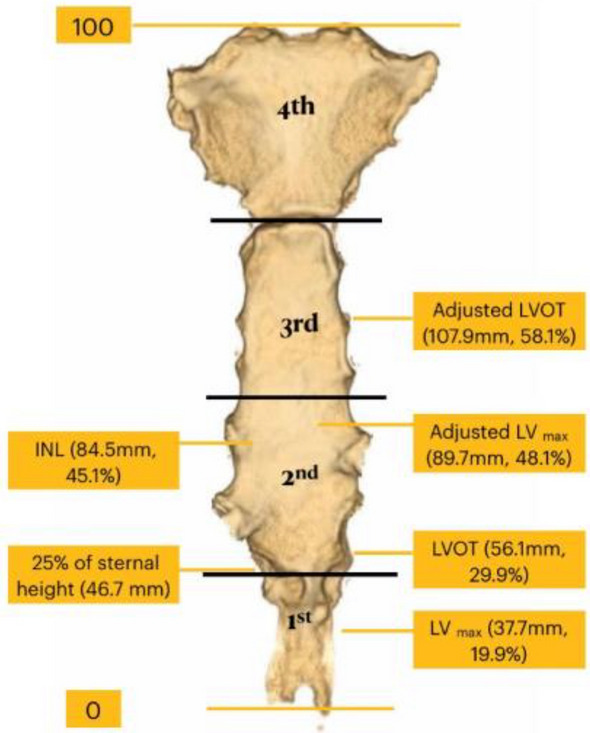
Distribution of reference body landmarks in 4 quarters of the sternum. INL: inter nipple line, LV_max_: The level of maximal width of the left ventricular base, LVOT: left ventricular outflow tract (Picture by NS. and Pitsucha S).

For reference purpose, we divided the sternum into quarters by height with respect to the tip of the xiphoid process. The distribution of each body landmark of interest in the four levels as Fig. 4 were shown in Table 3**.** The majority of the subjects had inter-nipple lines located within the lower half of the sternum, 145/204 (71.08%) within the 2nd quarter from the TOX. All of the LV_max_ were located in the lower half of the sternum (70.59% and 29.41% in the 1st and 2nd lower quarters of the sternum, respectively). The adjusted height of the LV_max_ was 89.7 mm which was at 48.1% of the sternal length, just below the midpoint of the sternal length within the 2nd quarter from TOX (Table 2**, **Figs. 3 and 4**)**. Almost all participants had the locationsof LVOT in the lower half of the sternum. The adjusted height of the LVOT was 107.9 mm (58.1% of sternal length) just above midpoint of the sternal length within the 3^rd^ quarter from TOX (Fig. 4). Of the 178 subjects whose uppermost part of the liver were above TOX, 80.34% were located in the 1st lower quarter of the sternum. Of the 109 subjects whose uppermost part of the stomach were above TOX, 94.50% were located within the 1st lower quarter of the sternum.

**Table 3:** Distribution of reference body landmarks in 4 quarters of the sternum.

Landmarks	Levels of sternum from tip of xiphoid	Overall (*n* = 204) N (%)	Gender		*P* value
			Female (*n* = 106)N (%)	Male (*n* = 98) N (%)	
Inter-nipple line	4th	0	0	0	0.95
	3th	56 (27.45)	28 (26.42)	28 (28.57)	
	2nd	145 (71.08)	76 (71.7)	69 (70.41)	
	1st	3 (1.47)	2 (1.89)	1 (1.02)	
LV_max_	4th	0	0	0	1
	3th	0	0	0	
	2nd	60 (29.41)	31 (29.25)	29 (29.59)	
	1st	144 (70.59)	75 (70.75)	69 (70.41)	
LVOT	4th	0	0	0	0.94
	3th	2 (0.98)	1 (0.94)	1 (1.02)	
	2nd	144 (70.59)	74 (69.81)	70 (71.43)	
	1st	58 (28.43)	31 (29.25)	27 (27.55)	
Uppermost liver	4th	0	0	0	1.00
(*n* = 178)	3th	1 (0.56)	1 (1.1)	0 (0)	
	2nd	34 (19.10)	17 (18.68)	17 (19.54)	
	1st	143 (80.34)	73 (80.22)	70 (80.46)	
Uppermost stomach	4th	0	0	0	1.00
(*n* = 109)	3th	0	0	0	
	2nd	6 (5.5)	3 (5.08)	3 (6)	
	1st	103 (94.5)	56 (94.9)	47 (94)	

LV_max_: The level of maximal width of the left ventricular base, LVOT: left ventricular outflow tract.

The distribution of each body landmark with respect to the midpoint of the lower half of the sternum (MLHS) and the distances from the reference point are shown in Table 4**.** Nearly all of the subjects (201/204 had inter-nipple lines located above MLHS with a median distance of 39.1 mm (IQR 28.2, 47.6) above the reference level. The LV_max_ were below MLHS in 144 (70.59%) subjects with a median distance of 15.8 mm (IQR 7.9, 23.4) below the reference level. The LVOT was located above MLHS in 146 (71.6%) participants with a median distance of 14.5 mm (IQR 7.6, 24.8). The uppermost part of the liver and stomach were mostly located below MLHS. The median distances from MLHS were 25.6 mm (IQR 16.3, 37.0) and 32.4 mm (IQR 22.8, 39.4), respectively.

**Table 4:** Position and distances (mm) of the reference body landmarks with respect to the midpoint of the lower half of the sternum (MLHS).

Landmarks	Position	Overall (*n* = 204) N (%)	Distance from MLHS in mm (IQR)
Inter-nipple line	Above	201 (98.53%)	39.1 (28.2, 47.6)
	Below	3 (1.47%)	14 (4.6, 33.3)
LV_max_	Above	60 (29.41%)	8.8 (4.6, 13.5)
	Below	144 (70.59%)	15.8 (7.9, 23.4)
LVOT	Above	146 (71.57%)	14.5 (7.6, 24.8)
	Below	58 (28.43%)	8.0 (3.9, 13.1)
Uppermost liver	Above	35 (19.66%)	10 (6.2, 23.5)
(*n* = 178)	Below	143 (80.34%)	25.6 (16.3, 37.0)
Uppermost stomach	Above	6 (5.50%)	10.4 (4.3, 15.0)
(*n* = 109)	Below	103 (94.50%)	32.4 (22.8, 39.4)

LV_max_: The level of maximal width of the left ventricular base, LVOT: left ventricular outflow tract, IQR: inter quartile range.

## Discussion

Direct ventricular compression is theoretically the most effective method for generating blood flow during CPR. Our primary concern is ensuring that the level of LV max targets a volume of cardiac output maximization while avoiding compression of outflow vessels^7^. Prior studies have shown that chest compressions focused on the left ventricle improve hemodynamics and has a greater likelihood of achieving return of spontaneous circulation (ROSC)^11^. Our results suggested that the optimal hand position, i.e., the adjusted height of LV_max_ for a Thai individual with expiration and arm down position (EAD) in this study group was at 89.7 mm above the tip of the xiphoid process which is approximately just below the midpoint of the sternum.

With concern of anatomical variants among populations, Lee et al.^9^ reported different heights and proportions of the LV_max_ to the sternal length between normal-weight and obese participants (27.7 mm versus 42.3 mm and 14.8% versus 22.0%, respectively). The mean height of the LV_max_ measured during inspiration and arm raised (IAR) position in our study group was at 37.7 mm above TOX which was in between the two aforementioned groups. This corresponds to the height at approximately 20% of sternal length in both genders. Similarly to our study, Shin et al.^12^ reported different absolute heights of maximal heart diameter cephalad to the xiphoid at 28 mm and 23 mm in male and female subjects, respectively. Even though the absolute heights were statistically different between male and female subjects, this difference is unlikely to be clinically significant due to the variation of adult rescuer palm heels’ width. The locations of LV_max_ during the IAR position were located within the lower half of the sternum for all subjects.

Nevertheless, during the actual CPR, patients are usually in arm down position with mostly expiration and sometimes inspiration in the case of assisted ventilation. The previous studies^9,12^ did not adjust their measurement for the limitation of the IAR position. In this study we attempted to adjust these measurements for the EAD position. According to Kwon et al.^8^, the LV_max_ levels during the EAD position were pulled more cephalad with average differences from the IAR position by 5.5 cm for males, 5.0 cm for females, and 5.2 cm for all patients, respectively. Therefore, the adjusted height of LV_max_ for EAD in our study group was estimated to be 89.7 mm from the tip of the xiphoid process. This corresponded to the 48% proportion of the sternal length which is just below the midpoint of the sternum and within the second quarter from TOX.

A few studies reported the optimal location of hand placement during CPR. In 2007, Shin et al. suggested, based on chest computed tomography (CT), that positioning the hands at the inter-nipple line mostly affected the root of the aorta, ascending aorta, and left ventricular outflow tract (LVOT) rather than the LV (20.6%)^12^. Similarly, in 2009, Hwang et al. applied trans-esophageal echocardiography (TEE) during CPR and found that the prior standard recommendation of hand position mostly affected the aorta (59%) and the LVOT (41%). In addition, 44% of their study population showed significant LVOT narrowing and subsequent reduction of stroke volume^13^. Kusunoki et al. also found that hand placement at the inter-nipple level resulted in the compression over the xiphoid process and epigastric region which may result in organ injuries^14^. Lee et al. found that the optimal chest compression position in both obese and normal weight individuals were more caudal than the “lower half of the sternum” which has been suggested since 2015 guideline^9^. Cha et al.^7^ conducted an analysis using chest CT scans on patients who had been resuscitated from cardiac arrest. Their findings indicated that chest compressions at the sternoxiphoid junction might offer enhanced effectiveness. In contrast, our results propose an optimal compression site at approximately 89.7 mm above the tip of xiphoid process just below midpoint of the sternum, ensuring compression on LV_max_ while minimizing the risk of intraabdominal organ injury.

Concerns for organ complications from chest compression includes compression on the LVOT and injuries to the liver and stomach^13,15,16^. Furthermore, rib and sternal fractures were the most common complication after CPR. Potentially lethal complications to abdominal organ such as laceration of liver, spleen and stomach were also reported at the recommended compression site^15,16^. However, it appears that there have been few case reports of intraabdominal organ injury during CPR, despite the fact that a significant portion of the liver and stomach is positioned in the lower region of the sternum^17,18^.

Hwang et al.^13^ revealed that the LVOT may be compressed during CPR resulting in varying degrees of LVOT narrowing and obstruction of blood flow from the left ventricle. In our study group, the height of the LVOT was in close proximity to the height of LV_max_, with adjusted LV_max_ and adjusted LVOT locating at about 89.7 mm and 107.9 mm from tip of xiphoid process, respectively. This would result in difficulty to avoid compressing to LVOT while placing rescuer’s hand over LV_max_ level. However, our study was performed using static CT data and is limited for demonstrating the hemodynamics of blood flow as well as morphologic change of the LVOT during CPR.

There were 87.3% (178/204) and 53.4% (109/204) of the subjects whose uppermost part of the liver and stomach were located in the thoracic region above the tip of xiphoid process with IAR position. Nearly all of these subjects had their uppermost part of the liver and stomach found in lower half of the sternum level in IAR position, majority in the 1st quarter from TOX. There is limited evidence in literature for adjusting the position of the intraabdominal organs during CPR from IAR position. However, assuming diaphragmatic elevation during expiration, majority of the subjects would have their liver and stomach located above tip of xiphoid process. This may support results of previous studies which reported liver and stomach laceration during CPR^16^. Based on our findings, it is likely more favorable to reduce the risk of abdominal organ injury by conducting chest compressions at the 2nd quarter of the sternum (at the level of the adjusted LV_max_) rather than at the 1st quarter from the tip of the xiphoid process. We recommend avoiding the 3^rd^ quarter of the sternum (corresponding to the adjusted LVOT level) to maximize stroke volume.Nevertheless, there is a need for observation of the dynamic change of the compressed structures during the actual CPR situation.

The 2005 edition of the AHA guideline for CPR previously recommended inter-nipple line level as the proper hand position for chest compression^6^. The later editions^3,4^ have changed the recommendation to the lower half of the sternum which is more ambiguous. This study showed that the mean height of the inter-nipple line was 84.5 mm from the tip of the xiphoid process, just below the midpoint of the sternum (45% of sternal length) in the IAR. Although there is insufficient evidence in the literature to adjust the inter-nipple line for the EAD position, we assumed that the inter-nipple should move slightly downward with the EAD position compared to the IAR position. Thus, the majority of the study group would have the inter-nipple line located within the lower half of the sternum during EAD and would therefore correspond with the current guideline^12,19^. Using the inter-nipple line to determine proper hand position may be more comprehensible to the general public in contrast to the ambiguous lower half of the sternum. According to the result of this study, it may be useful to educate non-healthcare rescuers to perform CPR using the more comprehensible inter-nipple line as the reference body landmark for hand compression.

There were several limitations in this study. The primary limitation of this study is that the participants were not individuals who had experienced cardiac arrests. In other words, since the study cohort differs from the population of cardiac arrest patients, it is important to exercise caution when generalizing the findings of this study to individuals who have suffered cardiac arrests. Second, this is a pilot study with a limited number of subjects as part of a multi-regional investigation. Further data collection of a larger group of subjects in different regions of the country would more closely represent Thai population. Nevertheless, the results could represent trends of optimal hand position during CC in Thai individuals. Third, the actual heights of anatomical landmarks during expiration and arm down position as of cardiac arrests’ postition were not determined due to the routine CT scan protocols in IAR position. However, we attempted to adjust these measurements (LV_max_ and LVOT levels) based on findings of previous study in Asian population to best represent the values found in CPR situation. Fourth, chest CT can be acquired at any phase of the cardiac cycle, which may result in variations in LV_max_ across different cardiac cycles. Fifth, about half of the patients had history of lung diseases. Some conditions such as emphysema and interstitial lung disease might affect the patient’s lung capacity and thoracic volume. Lastly, there was insufficient evidence to adjust the height of inter-nipple line with respect to the sternum in EAD position. It may be essential to further examine the position of inter-nipple level on Chest CT in IAR and EAD positions.

## Conclusion

The optimal hand position for chest compression (LV_max_) during CPR in Thai Ramathibodi population was approximately 37.7 mm (20% of sternal length) superior to the xiphoid process. After adjusting for the expired arm down position, this level moved upward to 89.7 mm (48% of sternal length) above TOX. Almost all the participants had the uppermost part of liver and stomach in the lower half of the sternum region. Hand position at the upper part of lower half of the sternum is closest to the LV_max_ and likely has a better chance to avoid compression intraabdominal organs.

### Supplementary Information

Supplementary Information.

## Data Availability

The data supporting this study’s findings are openly available in Harvard Dataverse at https://doi.org/10.7910/DVN/7DCDZK.

## References

[1] Redberg RF, Tucker KJ, Cohen TJ, Dutton JP, Callaham ML, Schiller NB (1993). Physiology of blood flow during cardiopulmonary resuscitation. Trans. Echocardiogr. Study Circ..

[2] Ewy GA (2018). The mechanism of blood flow during chest compressions for cardiac arrest is probably influenced by the patient's chest configuration. Acute Med. Surg..

[3] Panchal AR, Bartos JA, Cabañas JG, Donnino MW, Drennan IR, Hirsch KG (2020). Part 3: Adult basic and advanced life support: 2020 American heart association guidelines for cardiopulmonary resuscitation and emergency cardiovascular care. Circulation..

[4] Berg RA, Hemphill R, Abella BS, Aufderheide TP, Cave DM, Hazinski MF (2010). Part 5: Adult basic life support. Circulation..

[5] Kosmopoulos M, Kalra R, Bartos JA, Raveendran G, Yannopoulos D. Contemporary approaches to cardiopulmonary resuscitation: physiology-guided approaches. J. Emerg. Crit. Care Med. 4. (2019)

[6] Ecc Committee S (2005). Task forces of the American Heart A. 2005 American heart association guidelines for cardiopulmonary resuscitation and emergency cardiovascular care. Circulation..

[7] Cha KC, Kim YJ, Shin HJ, Cha YS, Kim H, Lee KH (2013). Optimal position for external chest compression during cardiopulmonary resuscitation: An analysis based on chest CT in patients resuscitated from cardiac arrest. Emerg. Med. J..

[8] Kwon H, Kim Y, Kim K, Jung JY, Kim J, Choi SI (2018). Where is the left ventricle during cardiopulmonary resuscitation based on chest computed tomography in the expiration with arms down position?. PLoS One..

[9] Lee J, Oh J, Lim TH, Kang H, Park JH, Song SY (2018). Comparison of optimal point on the sternum for chest compression between obese and normal weight individuals with respect to body mass index, using computer tomography: A retrospective study. Resuscitation..

[10] Wayne WD, Cross CL. Determination of sample size for estimating means. In: Wayne WD, Chad L. Cross, editor. Biostatistics: a foundation for analysis in the health sciences. Chapter 6: Estimation. 10th ed. ed. the United States of America: New York: Wiley & Sons; p. 189–91. (2013)

[11] Marshall RA, Morton JS, Luchkanych AMS, El Karsh Y, El Karsh Z, Morse C (2022). Left ventricle chest compression improves ETCO(2), blood pressure, and cerebral blood velocity in a swine model of cardiac arrest and cardiopulmonary resuscitation. Resusc. Plus..

[12] Shin J, Rhee JE, Kim K (2007). Is the inter-nipple line the correct hand position for effective chest compression in adult cardiopulmonary resuscitation?. Resuscitation..

[13] Hwang SO, Zhao PG, Choi HJ, Park KH, Cha KC, Park SM (2009). Compression of the left ventricular outflow tract during cardiopulmonary resuscitation. Acad. Emerg. Med..

[14] Kusunoki S, Tanigawa K, Kondo T, Kawamoto M, Yuge O (2009). Safety of the inter-nipple line hand position landmark for chest compression. Resuscitation..

[15] Krischer JP, Fine EG, Davis JH, Nagel EL (1987). Complications of cardiac resuscitation. Chest..

[16] Deliliga A, Chatzinikolaou F, Koutsoukis D, Chrysovergis I, Voultsos P (2019). Cardiopulmonary resuscitation (CPR) complications encountered in forensic autopsy cases. BMC Emerg. Med..

[17] Koutserimpas C, Ioannidis A, Siaperas P, Skarpas A, Tellos A, Velimezis G (2018). Intra-abdominal hemorrhage following cardiopulmonary resuscitation: A report of two cases. Case Rep. Emerg. Med..

[18] Graham B, Nutbeam T, Smith JE (2016). Abdominal trauma sustained during cardiopulmonary resuscitation may be detected by ultrasound. Trauma..

[19] Olszynski PA, Bryce R, Hussain Q, Dunn S, Blondeau B, Atkinson P (2021). A novel anatomic landmark to target the left ventricle during chest compressions in cardiac arrest. Cureus..

