# Supplementary material for: Optimal chest compression position for cardiopulmonary resuscitation determined by computed tomography image: retrospective cross-sectional analysis
Source: Sci Rep. 2023 Dec 20;13:22763. doi: 10.1038/s41598-023-49486-3 (PMC10733391; doi:10.1038/s41598-023-49486-3)
Supplement: Supplementary file 1 — Supplementary Information. [file 41598_2023_49486_MOESM1_ESM.docx]

**Supplement1**

**Proportion of the parameters of interest compared to the sternal length**

| **The interested parameters** | **Proportion to sternal length** |
| --- | --- |
| Proportion of INL to the SL (%) | $\frac{100 x INL}{SL}$ |
| Proportion of LV_max_ to the SL (%) | $\frac{100 x LVmax}{SL}$ |
| Proportion of LVOT to the SL (%) | $\frac{100 x LVOT}{SL}$ |
| Proportion of adjusted LV_max_ to the SL (%): | $\frac{100 x {adjusted \mathrm{LV}\max}}{SL}$ |
| Proportion of adjusted LVOT to the SL (%) | $\frac{100 x adjusted LVOT}{SL}$ |

INL: inter nipple line, SL: sternal length, LV_max_: The level of maximal width of the left ventricular base, LVOT: left ventricular outflow tract
